# Cu-MOF-Decorated 3D-Printed Scaffolds for Infection Control and Bone Regeneration

**DOI:** 10.3390/jfb16030083

**Published:** 2025-03-01

**Authors:** Ting Zhu, Qi Ni, Wenjie Wang, Dongdong Guo, Yixiao Li, Tianyu Chen, Dongyang Zhao, Xingyu Ma, Xiaojun Zhang

**Affiliations:** 1 School of Medicine, Northwest University, Xi’an 710069, China; 2 Key Laboratory of Resource Biology and Biotechnology Western China, Ministry of Education, Provincial Key Laboratory of Biotechnology, College of Life Sciences, Northwest University, Xi’an 710069, China

**Keywords:** copper, metal–organic frameworks, 3D printing scaffold, antibacterial properties, osteoinductive potential, bone regeneration

## Abstract

Infection control and bone regeneration remain critical challenges in bone defect treatment. We developed a 3D-printed scaffold incorporating copper-based metal–organic framework-74 (Cu-MOF-74) within a polycaprolactone/hydroxyapatite composite. The synthesized Cu-MOF-74 exhibited a well-defined crystalline structure and rod-like morphology, as confirmed by TEM, EDS, FTIR, and XRD analyses. The scaffolds exhibited hierarchical pores (100–200 μm) and demonstrated tunable hydrophilicity, as evidenced by the water contact angles decreasing from 103.3 ± 2.02° (0% Cu-MOF-74) to 63.60 ± 1.93° (1% Cu-MOF-74). A biphasic Cu^2+^ release profile was observed from the scaffolds, reaching cumulative concentrations of 98.97 ± 3.10 ppm by day 28. Antimicrobial assays showed concentration-dependent efficacy, with 1% Cu-MOF-74 scaffolds achieving 90.07 ± 1.94% and 80.03 ± 2.17% inhibition against *Staphylococcus aureus* and *Escherichia coli*, respectively. Biocompatibility assessments using bone marrow-derived mesenchymal stem cells revealed enhanced cell proliferation at Cu-MOF-74 concentrations ≤ 0.2%, while concentrations ≥ 0.5% induced cytotoxicity. Osteogenic differentiation studies highlighted elevated alkaline phosphatase activity and mineralization in scaffolds with 0.05–0.2% Cu-MOF-74 scaffolds, particularly at 0.05% Cu-MOF-74 scaffolds, which exhibited the highest calcium deposition and upregulation of bone sialoprotein and osteopontin expression. These findings demonstrate the dual functional efficacy of Cu-MOF-74/PCL/HAp scaffolds in promoting both infection control and bone regeneration. These optimized Cu-MOF-74 concentrations (0.05–0.2%) effectively balance antimicrobial and osteogenic properties, presenting a promising strategy for bone defect repair in clinical applications.

## 1. Introduction

Bone defects caused by trauma, infection, or tumor resection present a significant clinical challenge, often requiring complex interventions to achieve effective bone regeneration [1]. Annually, millions of patients worldwide undergo surgical procedures to address bone defects, leading to substantial social and economic burdens [2]. Autologous bone grafts, considered to be the gold standard for bone regeneration, are limited by donor site morbidity and insufficient material availability [3,4,5]. Allogeneic bone grafts, while a viable alternative, pose risks of immune rejection, disease transmission, and suboptimal integration with host tissues [4,6]. Synthetic bone substitutes, such as bone cements, are typically restricted to small, non-load-bearing defects due to their limited porosity and degradability, which are crucial for vascularization and long-term bone regeneration [7,8]. Therefore, the development of innovative therapeutic strategies that combine structural integrity, bioactivity, and antibacterial properties is urgently needed to overcome these limitations.

Tissue engineering has emerged as a promising strategy for bone defect repair, with scaffolds designed to mimic the natural bone microenvironment and promote regenerative processes. Scaffolds provide a three-dimensional (3D) architecture that supports cell adhesion, proliferation, and differentiation, while simultaneously delivering bioactive cues to enhance tissue regeneration [9,10,11,12]. Among the various scaffold fabrication techniques, 3D printing has gained significant attention due to its capacity to create complex, patient-specific structures with precise control over porosity, geometry, and material composition [12,13]. Polycaprolactone (PCL), a biodegradable polyester approved by the FDA for medical applications, is frequently employed in 3D printing owing to its favorable mechanical properties and processability [14]. However, PCL lacks intrinsic bioactivity, necessitating the incorporation of bioactive components such as hydroxyapatite (HAp) and metal ions to enhance its osteogenic potential [15].

To enhance the bioactivity of PCL scaffolds, HAp, a primary mineral component of natural bone, has been extensively utilized in bone tissue engineering due to its excellent biocompatibility and osteoconductive properties [16]. The incorporation of HAp into PCL scaffolds has been shown to enhance osteogenic activity and mechanical strength, rendering it a suitable composite for bone regeneration [15]. However, a critical limitation of HAp/PCL scaffolds is the absence of intrinsic antibacterial properties, which is a significant concern given the high risk of infection associated with bone defect repair, particularly in cases of trauma or complex fractures. Postoperative infections can severely compromise bone healing, leading to complications and implant failure [17]. To address this limitation, researchers have explored incorporating metal ions, such as copper (Cu^2+^), into scaffolds to impart both antibacterial and osteogenic functionalities [18,19,20].

Copper, an essential trace element, plays a crucial role in numerous physiological processes, including angiogenesis, enzymatic activity, and bone metabolism [21]. Recent studies have demonstrated that copper-doped scaffolds can enhance osteogenic differentiation and exhibit antibacterial activity, positioning them as promising candidates for bone defect repair [19,20]. However, uncontrolled copper release can induce cytotoxicity, highlighting the need for controlled release mechanisms to ensure therapeutic efficacy and safety [22]. Metal–organic frameworks (MOFs), particularly copper-based MOFs (Cu-MOFs), offer a compelling strategy by enabling the sustained release of Cu^2+^ while maintaining structural integrity and biocompatibility [23,24]. Cu-MOFs are porous crystalline materials composed of metal ions coordinated with organic ligands, providing a high surface area and tunable physicochemical properties [24]. Among Cu-MOFs, Cu-MOF-74 has attracted considerable interest due to its high density of open metal sites, which are known to enhance antibacterial and osteogenic properties [25,26]. The porous structure of Cu-MOF-74 facilitates controlled copper ion release, mitigating the risk of cytotoxicity associated with a burst release. Furthermore, the inherent properties of Cu-MOF-74 itself may contribute to enhanced biocompatibility and osteogenic potential within the scaffold. Given the critical challenge of bacterial infection in bone reconstruction—particularly in trauma settings, where compromised soft tissue and vascularization increase susceptibility to colonization [27]—the incorporation of Cu-MOF-74 into bone scaffolds represents a rational approach to address both regenerative and antimicrobial requirements.

In this study, we developed Cu-MOF-74/HAp/PCL composite scaffolds using 3D printing. We hypothesized that incorporating Cu-MOF-74 into HAp/PCL scaffolds would enhance their antibacterial and osteogenic properties, rendering them suitable for bone defect repair and infection control. The scaffolds were characterized for their structural, mechanical, and biological properties, and their antibacterial and osteogenic potential was evaluated in vitro. Our findings demonstrate that Cu-MOF-74/HAp/PCL scaffolds exhibit excellent biocompatibility, antibacterial activity, and osteogenic potential, offering a promising solution for bone defect repair and regeneration.

## 2. Materials and Methods

### 2.1. Materials

PCL was purchased from Engineering for Life Technology (Suzhou, China). HAp, monohydrated copper acetate (Cu(OAc)_2_·H_2_O), 2,5-dihydroxyterephthalic acid (DHTP), tetrahydrofuran (THF), and methanol were purchased from Macklin Biochemical Technology (Shanghai, China;. α-MEM medium was purchased from Hyclone Company, Logan, UT, USA. FITC-labeled phalloidin, Live/Dead Cell Staining Kits, Cell Counting Kit-8 (CCK-8), Alizarin Red S staining solution, BCA protein concentration test kits, and alkaline phosphatase (ALP) assay kits were provided by Beyotime Biotechnology (Shanghai, China). Fetal bovine serum (FBS) was provided by Cell-Box Biotechnology (Changsha, China). Primary antibodies, bone sialoprotein (BSP), osteopontin (OPN), and osteocalcin (OCN), were purchased from Proteintech Group (Wuhan, China).

### 2.2. Synthesis and Characterization of Cu-MOF-74

#### 2.2.1. Synthesis of Cu-MOF-74

Cu-MOF-74 was synthesized using a modified method reported by Zheng et al. [24]. Briefly, 0.4 g of Cu(OAc)_2_·H_2_O was dissolved in 10 mL of methanol to obtain a 40 g/L solution. Then, 0.2 g of DHTP was added to 5 mL of methanol, followed by the dropwise addition of the Cu(OAc)_2_·H_2_O solution. The mixture was magnetically stirred for 24 h, and the resulting reddish-brown crystalline solid was collected by filtration. The resulting Cu-MOF-74 was washed with methanol four times to remove impurities and dried in a vacuum at 85 °C for 24 h.

#### 2.2.2. Morphological Characterization of Cu-MOF-74

The microstructure of Cu-MOF-74 was observed using scanning electron microscopy (SEM) (Hitachi, S-4800, Tokyo, Japan) and transmission electron microscopy (TEM) (JEOL, JEM-2100Plus, Tokyo, Japan).

#### 2.2.3. Physicochemical Characterization of Cu-MOF-74

The crystal structures of the synthesized nanoparticles were analyzed using X-ray diffraction (XRD) (Bruker-D8, Bremen, Germany). Fourier-transform infrared (FTIR) spectra (AVATAR-FTIR-360, Thermo Fisher Scientific, Waltham, MA, USA) were recorded in the range of 4000–400 cm^−1^. Elemental mapping of Cu-MOF-74 was conducted using an energy-dispersive spectrometer (EDS) (Bruker QUANTAX 400, Bremen, Germany).

### 2.3. Fabrication and Characterization of Cu-MOF-74/HAp/PCL Scaffolds

#### 2.3.1. Fabrication of the Scaffolds

To prepare the printing bio-ink, 20 g of PCL and 4 g of HAp were dispersed in 200 mL of THF. Subsequently, Cu-MOF-74 was incorporated into the mixture at concentrations of 0%, 0.05%, 0.1%, 0.2%, 0.5%, and 1% by weight relative to PCL (Table 1). After thorough mixing to ensure homogeneity, the resulting blends were dried in a fume hood to remove the solvent, cut into small pieces, loaded into a printing cartridge, and melted at 65 °C within the printer. The scaffolds were printed using an extrusion-based pneumatic 3D printer (Engineering For Life Technology, Suzhou, China) with the following parameters: 6–10 MPa air pressure, 0.35 mm nozzle diameter, 30 mm/s printing speed, 65–70 °C extrusion temperature, 20 °C platform temperature, and 0.2 mm layer height. The scaffolds were designed using Bioplotter CAD/CAM software (EFL_PotatoE 6601 V1.2.6) and printed layer by layer with a pore size of 100 μm, 5 layers, and dimensions of 10 × 10 × 1 mm^3^.

#### 2.3.2. Structural Properties of the Scaffolds

The scaffolds were coated with gold and imaged using a field-emission scanning electron microscope (JEOL, JSM-7610Fplus, Tokyo, Japan).

#### 2.3.3. Hydrophilicity of the Scaffold Surfaces

The hydrophilicity of the scaffolds was evaluated by measuring the static water contact angle. Deionized water (20 μL) was dropped onto the scaffold surface, and the contact angle was calculated using imaging software (Java 1.8.0_322).

#### 2.3.4. Cu^2+^ Release Performance

The release dynamics of Cu^2+^ from the Cu-MOF-74/HAp/PCL composite scaffolds were quantified using inductively coupled plasma mass spectrometry (ICP-MS). The scaffolds were immersed in phosphate-buffered saline (PBS) at a ratio of 0.5 cm^2^/mL and incubated for 28 days. At predetermined timepoints (1, 4, 7, 14, 21, and 28 days), 3 mL of PBS supernatant was collected, and 3 mL of fresh PBS was added to each sample. The Cu^2+^ concentrations were determined via ICP-MS to construct ion release profiles.

### 2.4. Antibacterial Performance Testing

The antibacterial efficacy of the Cu-MOF-74/HAp/PCL composite scaffolds was assessed against a Gram-positive bacterium, *Staphylococcus aureus* (*S. aureus*, ATCC 25923), and a Gram-negative bacterium, *Escherichia coli* (*E. coli*, ATCC 25922). Specifically, 500 μL of the bacterial suspension, diluted to a concentration of 1 × 10^5^ CFU/mL, was added onto the sterilized Cu-MOF-74/HAp/PCL composite scaffolds, placed in a 24-well plate, and incubated at 37 °C for 12 h. Non-adherent bacteria were rinsed using PBS. Ultrasonic treatment (40 Hz, 10 min) was applied to dislodge bacteria adhering to the composite scaffolds. The bacterial solutions were diluted 1000 times, and 60 μL of the diluted solution was evenly spread on LB solid medium and incubated for another 12 h. The colony counts on the LB agar plates indirectly quantified the bacterial CFUs on the scaffold surfaces. The bacterial survival rate was calculated as follows:

$$R=\frac{B-A}{B}\times 100\%,$$

where

- A represents the average number of surviving bacteria on the Cu-MOF-74/HAp/PCL scaffolds;
- B represents the average number on 0 Cu scaffolds.

### 2.5. Cell Biocompatibility Analysis

#### 2.5.1. Cell Culture

Primary bone marrow mesenchymal stem cells (BMSCs) were obtained from one-week-old Sprague-Dawley rats. All animal experiments were performed in accordance with the relevant regulations of the Ministry of Health of the People’s Republic of China and the Guidelines on the Good Treatment of Laboratory Animals. The experiments were reviewed and approved by the Laboratory Animal Ethics Review Committee of Northwest University, China. Femurs and tibias were isolated using ophthalmic scissors and tweezers, with muscle and connective tissue removed. After rinsing with PBS, the bone marrow was flushed with α-MEM medium supplemented with 10% FBS and 1% penicillin–streptomycin to create a cell suspension. The suspension was then cultured in flasks at 37 °C under 5% CO_2_.

#### 2.5.2. Cell Attachment Assay

The growth and spreading morphology of BMSCs were characterized using F-actin staining. Briefly, the scaffolds were seeded with 5 × 10^4^ BMSCs and incubated at 37 °C for 1 day. After washing with PBS, the scaffolds were fixed in 4% paraformaldehyde overnight. The samples were permeabilized with 0.1% Triton X-100 for 15 min, stained with FITC-labeled phalloidin solution for 30 min, and observed using a Zeiss LSM 710 confocal microscope (Carl Zeiss AG, Oberkochen, Germany).

#### 2.5.3. Cell Viability Analysis

BMSCs were seeded onto the Cu-MOF-74/HAp/PCL composite scaffolds at a concentration of 1 × 10^5^ cells per scaffold. After 3 d of incubation, the cells were treated with live/dead cell staining solution for 30 min, washed three times with PBS, and subsequently observed using laser confocal microscopy. Live cells fluoresced green, while dead cells fluoresced red.

#### 2.5.4. Cell Proliferation Assay

BMSCs were cultured on Cu-MOF-74/HAp/PCL composite scaffolds at a density of 1 × 10^5^ cells per scaffold for 7 d. At predetermined time intervals (days 1, 4, and 7), the scaffolds were aseptically transferred to new 24-well plates, and 500 μL of working solution containing 10% CCK-8 reagent and 90% cell culture medium was added. Following 2 h of incubation, 100 μL of supernatant from each sample was transferred to a 96-well plate. Absorbance measurements were performed at a wavelength of 450 nm using a microplate reader (BioTek Synergy H1, Agilent, Santa Clara, CA, USA).

### 2.6. Osteogenic Differentiation Analysis

#### 2.6.1. Cellular ALP Activity Assay

BMSCs (1 × 10^5^ cells per scaffold) were cultured on Cu-MOF-74/HAp/PCL composite scaffolds with α-MEM medium supplemented with 10% FBS and 1% penicillin–streptomycin. At days 7 and 14 post-induction, the cells were washed twice with PBS, lysed with RIPA buffer, and quantified using an ALP assay kit according to the manufacturer’s protocol. Briefly, 50 μL of cell lysate was mixed with 100 μL of p-nitrophenyl phosphate (pNPP) substrate and incubated at 37 °C for 30 min. The reaction was terminated with 50 μL of 0.1 N NaOH, and absorbance was measured at 405 nm using a microplate reader (BioTek Synergy H1).

#### 2.6.2. Mineralization Assessment by Tetracycline Labeling

To evaluate extracellular matrix mineralization, BMSC-seeded scaffolds (1 × 10^5^ cells per scaffold) were cultured for 21 d, with the medium replaced every 3 d. The samples were stained with 5 mg/mL tetracycline hydrochloride for 1 h at 37 °C, followed by three washes with PBS. Fresh α-MEM medium was added, and the samples were further incubated for 1 h at 37 °C under 5% CO_2_. After fixation with 4% paraformaldehyde for 30 min, the mineralization patterns were visualized using a confocal laser scanning microscope (Leica, TCS SP8 MP, Heidelberg, Germany).

#### 2.6.3. Calcium Deposition Quantification via Alizarin Red S Staining

After 21 d of incubation, scaffolds were fixed with 4% paraformaldehyde overnight at 4 °C and stained with 2% Alizarin Red S for 30 min. Excess dye was removed by five sequential PBS washes under gentle agitation. For quantification, mineralized nodules were dissolved in 10% cetylpyridinium chloride in 10 mM sodium phosphate buffer for 30 min. The solution was centrifuged at 11,000 rpm for 5 min, and the absorbance of the supernatant was measured at 562 nm.

#### 2.6.4. Osteogenic Protein Expression Profiling

BMSCs were cultured onto Cu-MOF-74/HAp/PCL scaffolds for 21 d. The scaffolds were rinsed three times with PBS, sectioned, and placed in 1.5 μL EP tubes. Total cellular proteins were extracted using RIPA buffer containing 1% protease inhibitor on ice for 5 min, and then centrifuged at 12,000 rpm (4 °C) for 60 s. The protein concentration was determined using a BCA protein assay kit. Proteins were separated by 10% SDS-PAGE and transferred to PVDF membranes. Membranes were blocked with 5% non-fat dry milk in Tris-buffered saline with 0.1% Tween-20 for 2 h, and then incubated with primary antibodies against BSP, OPN, OCN, and β-tubulin overnight at 4 °C. After incubation with HRP-conjugated secondary antibodies for 1 h, the protein bands were detected and quantified with Image Lab Software (Java 1.8.0_322).

### 2.7. Statistical Analysis

The experiments were independently replicated a minimum of three times, and the data are presented as the mean ± standard deviation. Data were analyzed using one-way analysis of variance (ANOVA) utilizing GraphPad Prism 8.2.1. A *p*-value of less than 0.05 was considered statistically significant (* *p* < 0.05; ** *p* < 0.01).

## 3. Results

### 3.1. Structure and Physical Characteristics of Cu-MOF-74

**Figure 1:**
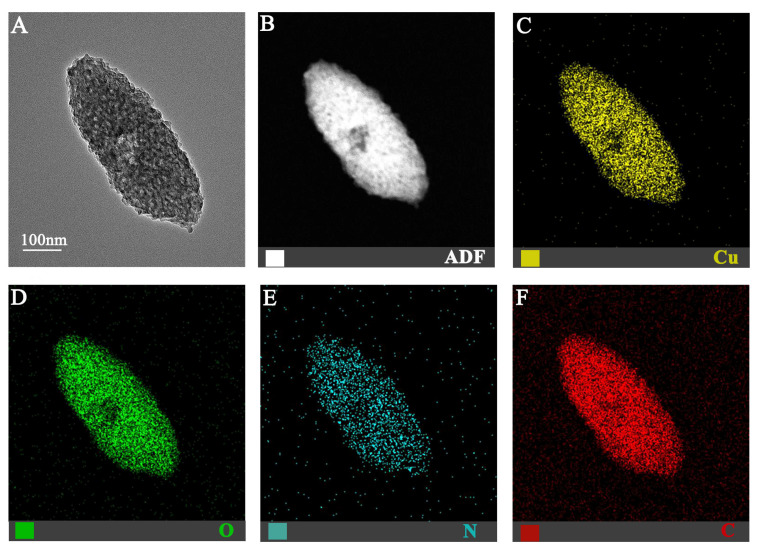
TEM image of Cu-MOF-74 (**A**) and the corresponding elemental mapping (**B**–**F**).

The morphological and structural properties of the synthesized Cu-MOF-74 were systematically characterized using advanced analytical techniques. TEM analysis revealed well-defined crystalline structures with uniform rod-like morphologies, measuring 300–500 nm in length and 50–100 nm in width (Figure 1A). EDS elemental mapping (Figure 1B–F) demonstrated a homogeneous distribution of constituent elements, with mass percentages of 64.54% C, 0.94% N, 18.20% O, and 16.33% Cu, and corresponding atomic percentages of 78.62% C, 0.98% N, 16.64% O, and 3.76% Cu. These values align closely with the theoretical stoichiometry of Cu-MOF-74, confirming the successful synthesis of the nanoscale metal–organic framework.

**Figure 2:**
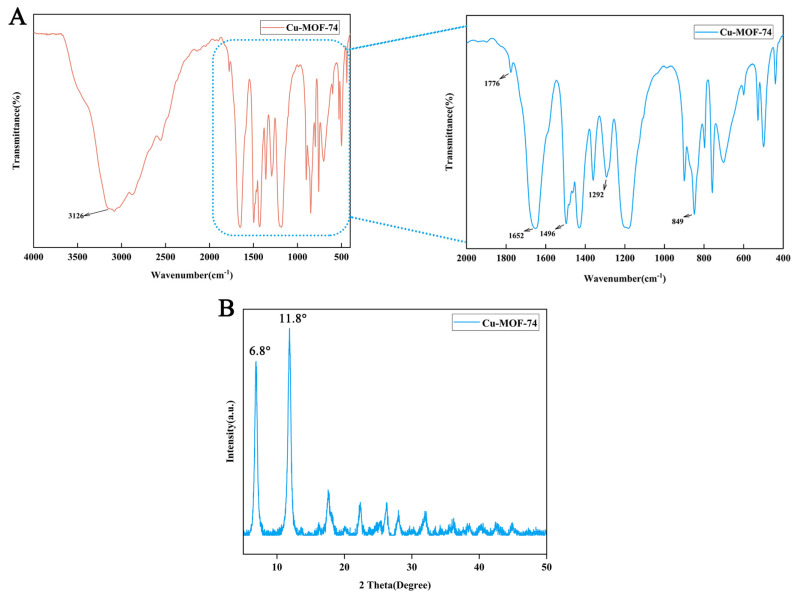
FTIR spectra (**A**) and XRD pattern (**B**) of Cu-MOF-74.

The FTIR spectrum, as depicted in Figure 2A, exhibited a broad absorption band in the range of 3000–3650 cm^−1^, attributed to O-H stretching vibrations from coordinated water molecules and free hydroxyl groups. Characteristic peaks were observed at 1292 cm^−1^ (C-O stretching of carboxyl groups), 1496 cm^−1^ (C=O stretching), and 1774 cm^−1^ (O-H bending vibrations), indicating the successful coordination between the -COOH groups of DHTP and Cu^2+^ ions. A prominent peak at 1652 cm^−1^ was assigned to the asymmetric stretching of -COO^−^ groups in the organic ligand, while the peak at 849 cm^−1^ corresponded to C-H bending vibrations in the aromatic ring of DHTP. Notably, a low-frequency absorption peak near 500 cm^−1^ was identified as the Cu-O stretching vibration, providing direct evidence of the metal–ligand coordination bond formation.

XRD analysis further confirmed the crystalline structure of Cu-MOF-74 (Figure 2B). The diffraction pattern exhibited two distinct and sharp peaks at 2θ = 6.8° and 11.8°, corresponding to the (001) and (200) crystal planes, respectively. These diffraction features validate the successful synthesis of Cu-MOF-74 with high crystallinity.

### 3.2. Structure and Physical Characteristics of the 3D-Printed Cu-MOF-74/PCL/HAp Composite Scaffolds

**Figure 3:**
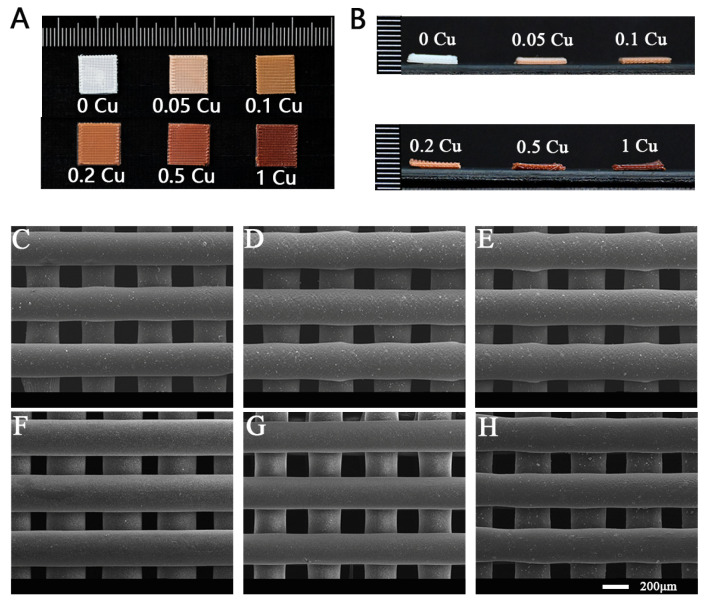
Photographs of the Cu-MOF-74/HAp/PCL composite scaffolds (**A**,**B**) and SEM images of the different Cu-MOF-74/HAp/PCL scaffolds: 0 Cu (**C**), 0.05 Cu (**D**), 0.1 Cu (**E**), 0.2 Cu (**F**), 0.5 Cu (**G**), and 1 Cu (**H**).

The 3D-printed Cu-MOF-74/PCL/HAp composite scaffolds exhibited a rectangular geometry (10 × 10 × 1 mm^3^) and a progressive color transition from white (0 Cu) to light pink (0.05 Cu) and, ultimately, to brownish-red (1 Cu) with increasing Cu-MOF-74 concentrations, while the macroscopic morphology remained unchanged (Figure 3A,B). The well-defined grid-like architecture showed uniform filament diameters and hierarchically interconnected pores (100–200 μm), as confirmed by SEM (Figure 3C–H). SEM analysis revealed smooth, compact surfaces at lower concentrations of Cu-MOF-74 (<0.2%), suggesting effective encapsulation of Cu-MOF-74 particles by the PCL/HAp matrix. Higher Cu-MOF-74 concentrations (≥0.5%) induced modest surface roughening, potentially enhancing cell adhesion through topographical cues.

**Figure 4:**
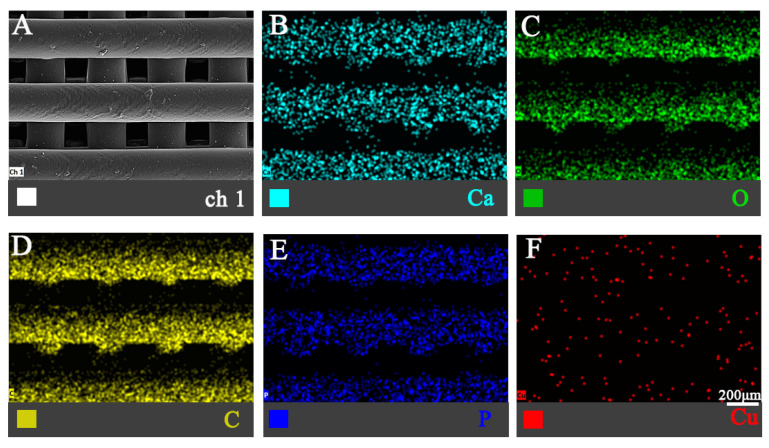
EDS mapping of the 0.2% Cu-MOF-74/HAp/PCL composite scaffolds: ch1 (**A**), Ca (**B**), O (**C**), C (**D**), P (**E**), and Cu (**F**).

EDS mapping (Figure 4) verified the homogeneous elemental distribution of C (54.76 ± 5.18%), O (36.21 ± 3.50%), Ca (6.15 ± 0.32%), P (2.71 ± 0.12%), and Cu (0.17 ± 0.02%) in the 0.2 Cu composite scaffolds, confirming successful composite integration without phase segregation.

**Figure 5:**
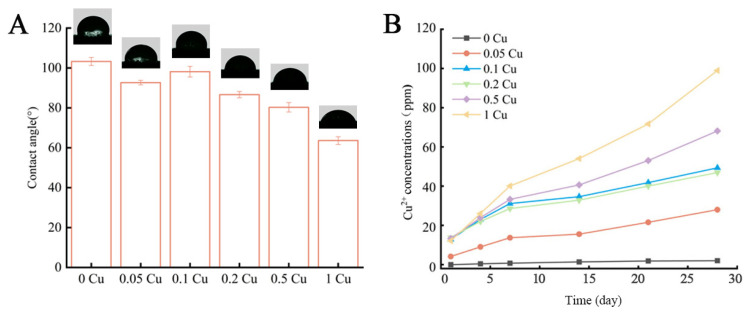
Water absorption process of Cu-MOF-74/HAp/PCL (**A**) and released Cu^2+^ concentration of the Cu-MOF-74/HAp/PCL immersed in PBS solution (**B**).

The hydrophilicity of the composite scaffolds showed significant Cu-MOF-74 dose-dependence (*p* < 0.01). The water contact angles decreased from 103.3 ± 2.02° (0 Cu) to 63.60 ± 1.93° (1 Cu) (Figure 5A), transitioning from hydrophobic to hydrophilic behavior (threshold at 0.2 Cu). This enhancement in hydrophilicity correlates with the increased surface polarity imparted by the coordinatively unsaturated metal sites of Cu-MOF-74.

The Cu^2+^ release kinetics demonstrated a biphasic pattern: an initial burst release (days 1–7), followed by a sustained release (days 7–28) (Figure 5B). During the first 7 d, rapid dissolution of Cu^2+^ from the scaffold surfaces was observed. From day 7 onward, the release rate decelerated, entering a transient plateau phase. The release persisted until day 28 and potentially beyond. The cumulative release at day 28 reached 28.02 ± 1.3 ppm (0.05 Cu), 49.33 ± 2.1 ppm (0.1 Cu), 46.93 ± 1.8 ppm (0.2 Cu), 68.16 ± 2.4 ppm (0.5 Cu), and 98.97 ± 3.1 ppm (1 Cu), showing a strong positive correlation with Cu-MOF-74 loading.

### 3.3. Antibacterial Properties of Cu-MOF-74/PCL/HAp Composite Scaffolds

**Figure 6:**
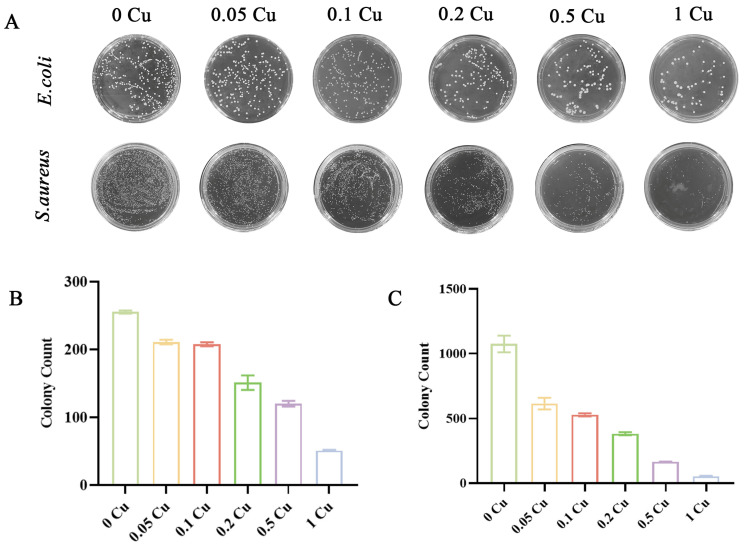
Optical images (**A**) and graphs of the percentage inhibition of colony counts for Cu-MOF-74/HAp/PCL scaffolds against *E. coli* (**B**) and *S. aureus* (**C**) at different concentrations.

The antimicrobial efficacy of the 3D-printed Cu-MOF-74/PCL/HAp scaffolds was quantitatively assessed against two clinically relevant bacterial strains, *S. aureus* (Gram-positive) and *E. coli* (Gram-negative), using the standardized colony-forming unit counting method. As illustrated in Figure 6, a clear concentration-dependent antibacterial relationship was observed.

Scaffolds containing 0.2% Cu-MOF-74 demonstrated significant antimicrobial activity, achieving inhibition rates of 64.43 ± 3.21% against *S. aureus* and 40.73 ± 2.85% against *E. coli*. Notably, increasing the Cu-MOF-74 loading to 1% substantially enhanced these values to 90.07 ± 1.94% and 80.03 ± 2.17% for *S. aureus* and *E. coli*, respectively (*p* < 0.01). This pronounced enhancement suggests synergistic effects between copper ion release and scaffold surface modification. The corresponding antibacterial rates are summarized in Table 2.

### 3.4. Biocompatibility and Proliferation of BMSCs on Cu-MOF-74/HAp/PCL Composite Scaffolds

**Figure 7:**
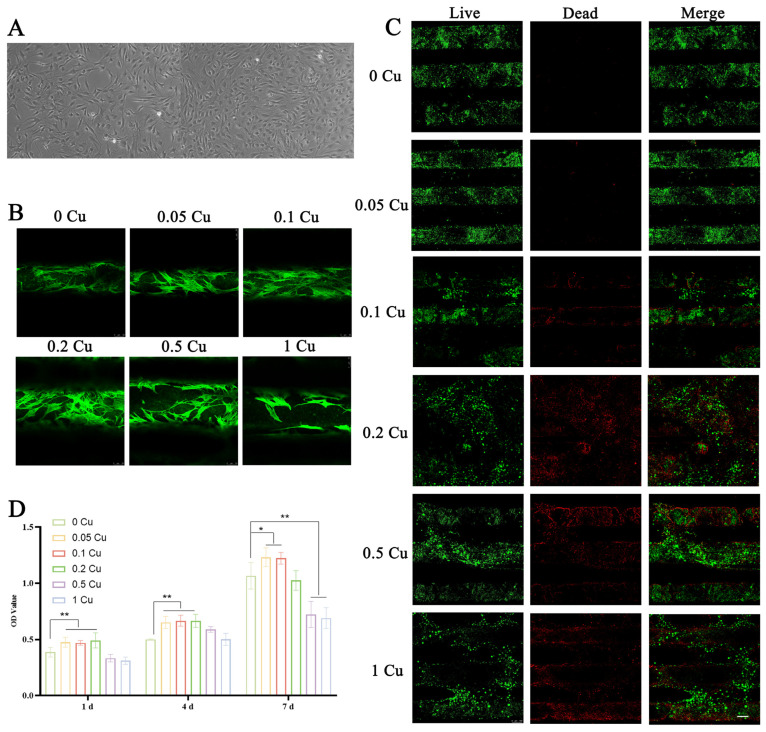
Images of BMSCs extracted from rat bone marrow (**A**). Cytoskeleton staining of BMSCs grown on different scaffolds for 3 d (**B**). Live/dead staining of BMSCs grown on different scaffolds for 3 d (**C**). The proliferation of BMSCs on different composite scaffolds for 1, 4, and 7 d (**D**). * *p* < 0.05, ** *p* < 0.01.

Primary BMSCs displayed short spindle and polygonal morphologies, exhibited strong adhesion properties, and demonstrated good refractivity (Figure 7A). Confocal microscopy of cytoskeletal staining revealed that BMSCs adhered effectively to the scaffold surfaces, with cells spreading uniformly across the scaffolds. Notably, BMSCs extended pseudopodia to anchor onto the scaffold, suggesting favorable cell–scaffold interactions (Figure 7B).

To further assess cell viability, live/dead staining was performed on scaffold-encapsulated BMSCs after three days of culture. As shown in Figure 7C, 80% of the images displayed green fluorescence, indicating high cell viability. The 0 Cu group exhibited negligible red fluorescence, signifying minimal cell death, while the other groups also showed limited red fluorescence, confirming the excellent biocompatibility of the Cu-MOF-74/PCL/HAp composite scaffolds. However, the 0.2 Cu, 0.5 Cu, and 1 Cu groups displayed increased red fluorescence, suggesting higher rates of cell death, which may be attributable to the cytotoxic effects of elevated copper concentrations.

The CCK-8 assay results showed that the 0.05 Cu, 0.1 Cu, and 0.2 Cu groups significantly promoted BMSC proliferation compared to the control group at 1 and 4 days of culture (*p* < 0.01). Over a 7-day culture period, lower copper concentrations (≤0.2 Cu) showed no significant cytotoxicity, with the 0.05 Cu, 0.1 Cu, and 0.2 Cu groups significantly promoting BMSC proliferation (*p* < 0.05). In contrast, the 0.5 Cu and 1 Cu groups exhibited significant inhibition of cell proliferation (*p* < 0.01), likely due to the higher Cu-MOF-74 content in the scaffolds, which may interfere with cell growth (Figure 7D).

### 3.5. Osteogenic Differentiation and Mineralization of BMSCs on Cu-MOF-74/HAp/PCL Composite Scaffolds

**Figure 8:**
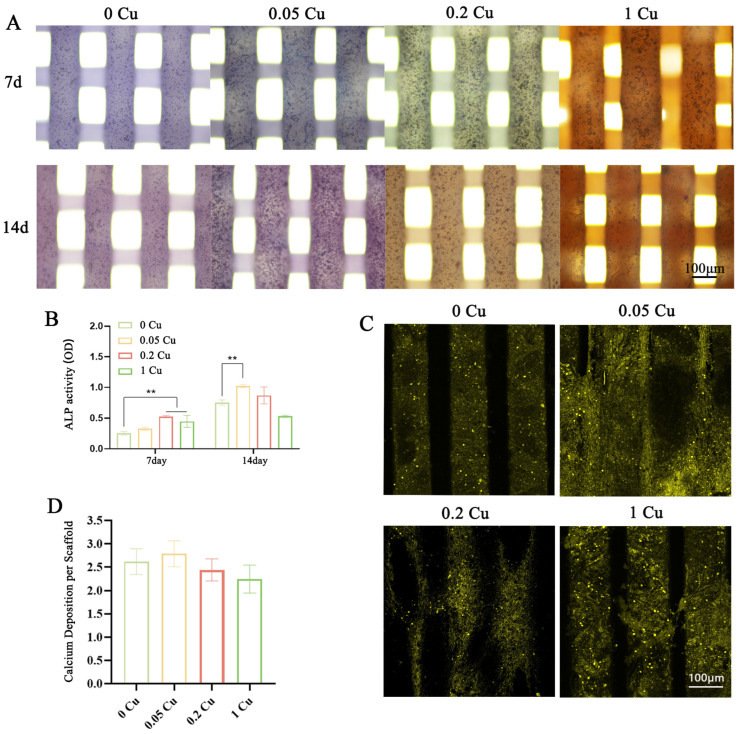
ALP staining (**A**) and ALP quantitative analysis (**B**) at 7 d and 14 d, and tetracycline staining (**C**) and Alizarin Red staining quantitative analysis (**D**) for 21 d; ** *p* < 0.01.

To systematically evaluate the osteogenic potential of the Cu-MOF-74/HAp/PCL composite scaffolds, ALP activity and mineralization capacity were analyzed at multiple timepoints. ALP staining and quantitative analysis revealed distinct temporal and concentration-dependent effects on BMSC differentiation. At day 7, ALP staining showed significantly more intense dark blue coloration in the 0.2 Cu and 1 Cu composite scaffolds compared to the control group (0 Cu), indicating higher ALP activity (Figure 8A). Quantitative analysis further confirmed that the 0.2 Cu and 1 Cu groups exhibited 2.1-fold and 1.8-fold higher ALP activity, respectively, than the control group (*p* < 0.01). Notably, the 0.2 Cu group demonstrated the highest ALP activity at this timepoint (Figure 8B). By day 14, the 0.05 Cu group showed the most pronounced ALP activity, with a 1.4-fold increase compared to the control (*p* < 0.01). However, higher copper concentrations (≥0.2 Cu) did not significantly promote ALP activity (*p* > 0.05).

Mineralization assays were performed at day 21 to evaluate the late-stage osteogenic differentiation of BMSCs. Tetracycline staining revealed stronger fluorescence intensity in the 0.05 Cu group, indicating more abundant calcium deposition compared to the control group (Figure 8C). Similarly, quantitative analysis of Alizarin Red S staining showed higher color intensity in the 0.05 Cu group (Figure 8D). However, there were no significant differences between the Cu-MOF-74-containing groups and the control group.

**Figure 9:**
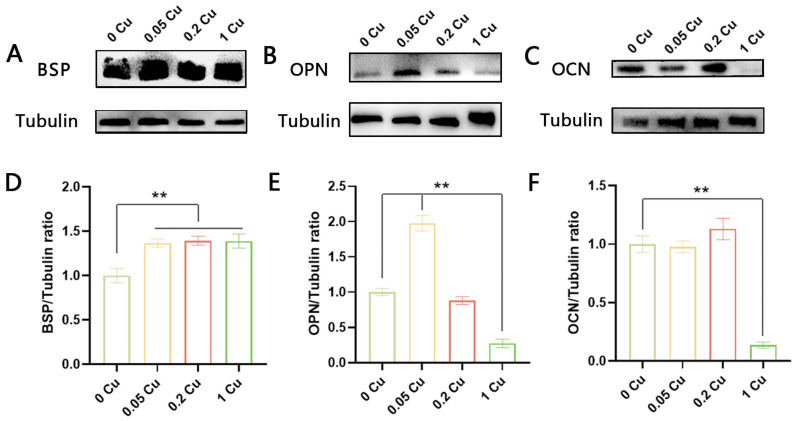
Western blot analysis of osteogenic marker expression (**A**–**D**) and quantitative analysis (**D**–**F**) at 21 days; (**A**,**D**) bone sialoprotein (BSP), (**B**,**E**) osteopontin (OPN), (**C**,**F**) osteocalcin (OCN); β-tubulin was used to normalize protein input and quantification; ** *p* < 0.01.

To further explore the osteogenic potential of Cu-MOF-74/HAp/PCL composite scaffolds, Western blot analysis was performed to assess the expression of osteogenesis-related proteins. BSP levels were significantly upregulated in all Cu-MOF-74-containing composite scaffolds compared to the control (*p* < 0.01) (Figure 9A,D). The 0.05 Cu group showed significantly elevated levels of OPN expression, with a 2.0-fold increase compared to the control (*p* < 0.01) (Figure 9B,E). In contrast, the 0.2 Cu group exhibited the highest expression of OCN. Notably, the 1 Cu group showed significantly decreased OCN expression (*p* < 0.01) (Figure 9C,F).

## 4. Discussion

The development of multifunctional scaffolds that combine antibacterial and osteogenic properties represents a significant advancement in bone tissue engineering. In this study, we successfully fabricated Cu-MOF-74/HAp/PCL composite scaffolds using 3D printing technology and demonstrated their potential for bone defect repair and infection control. The incorporation of Cu-MOF-74 into HAp/PCL scaffolds not only enhanced their antibacterial properties but also promoted the osteogenic differentiation of bone marrow-derived mesenchymal stem cells (BMSCs), making them ideal candidates for clinical applications.

The structural characterization of the scaffolds revealed a uniform porous architecture with interconnected pores ranging from 100 to 200 μm, which is optimal for cell adhesion, nutrient diffusion, and vascularization [28]. The addition of Cu-MOF-74 increased the hydrophilicity of the scaffolds, as evidenced by the reduction in water contact angles, a property known to enhance cell adhesion and proliferation [29]. The sustained release of Cu^2+^ ions from the scaffolds was observed over 28 days, with an initial burst release followed by a gradual release phase. This controlled release mechanism is critical for maintaining therapeutic copper concentrations while minimizing cytotoxicity [23,30]. The release kinetics of Cu^2+^ was influenced by the degradation of the PCL matrix and the hydrolysis of Cu-MOF-74, which is consistent with previous studies on MOF-based drug delivery systems [31].

The antibacterial properties of the scaffolds were evaluated against *S. aureus* and *E. coli*, two common pathogens associated with bone infections. The results demonstrated a concentration-dependent antibacterial effect, with the 1 Cu composite scaffolds showing a 90.07% reduction in *S. aureus* and an 80.03% reduction in *E. coli*. The antibacterial mechanism of Cu^2+^ ions involves the disruption of bacterial cell membranes, generation of reactive oxygen species, and interference with DNA and protein functions [32,33]. These findings are consistent with previous studies demonstrating the potent antibacterial activity of copper-doped biomaterials [19,20,34]. However, it is important to note that excessive copper release can lead to cytotoxicity, as observed in the 0.5% and 1% Cu-MOF-74 groups, which exhibited reduced cell viability and proliferation. Therefore, optimizing the concentration of Cu-MOF-74 is critical for balancing antibacterial activity and biocompatibility.

The osteogenic potential of the scaffolds was evaluated through ALP activity, calcium nodule formation, and the expression of osteogenic markers (BSP, OPN, and OCN). The 0.2% Cu-MOF-74 group exhibited the highest ALP activity and calcium deposition, indicating enhanced osteogenic differentiation of BMSCs. These findings are consistent with those of previous studies demonstrating the osteogenic effects of Cu^2+^, which are known to promote angiogenesis and bone formation [35,36]. The upregulation of osteogenic markers, particularly BSP and OPN, further supports the role of Cu-MOF-74 in promoting bone regeneration. However, the 1% Cu-MOF-74 group showed reduced osteogenic activity, likely due to the cytotoxic effects of high copper concentrations. This highlights the importance of optimizing copper release to achieve therapeutic efficacy without compromising cell viability.

The multifunctionality of Cu-MOF-74/HAp/PCL scaffolds can be attributed to the synergistic effects of HAp and Cu-MOF-74. HAp provides a bioactive surface that promotes cell adhesion and osteogenic differentiation, while Cu-MOF-74 imparts antibacterial properties and enhances angiogenesis. These findings align with recent reports that Cu^2+^ ions promote angiogenesis [37,38]. The combination of these components in a 3D-printed scaffold offers a promising solution for bone defect repair, particularly in infected or compromised bone sites. However, further studies are needed to evaluate the long-term in vivo performance of these scaffolds, including their degradation kinetics, mechanical stability, and ability to promote vascularized bone regeneration.

In conclusion, our study demonstrates the potential of Cu-MOF-74/HAp/PCL composite scaffolds for bone defect repair and infection control. Optimized concentrations of Cu-MOF-74 (0.05–0.2%) can effectively balance antimicrobial and osteogenic properties, presenting a promising strategy for clinical applications in bone defect repair. While these in vitro assays highlight the osteogenic potential of Cu-MOF-74/HAp/PCL scaffolds and their capacity to control infection and promote bone regeneration, further research is necessary to evaluate their long-term in vivo performance. Future studies should focus on assessing degradation kinetics, mechanical stability, and the ability of these scaffolds to promote vascularized bone regeneration in vivo, alongside continued optimization of Cu-MOF-74 concentrations and comprehensive evaluation in animal models.

## Figures and Tables

**Table 1:** List of compositions of 3D-printed scaffolds.

Name	Weight			Cu-MOF-74/PCL (*w*/*w*%)
	PCL (g)	HAp (g)	Cu-MOF-74 (g)	
0 Cu	20	4	0	0
0.05 Cu	20	4	0.01	0.05
0.1 Cu	20	4	0.02	0.1
0.2 Cu	20	4	0.04	0.2
0.5 Cu	20	4	0.1	0.5
1 Cu	20	4	0.2	1

**Table 2:** List of the bacterial survival rates for 3D-printed scaffolds.

Name	Bacterial Survival Rate (%)	
	*E. coli*	*S. aureus*
0 Cu	0	0
0.05 Cu	17.36 ± 1.36	42.70 ± 4.17 *
0.1 Cu	18.67 ± 1.13	50.79 ± 1.13 **
0.2 Cu	40.73 ± 2.85 *	64.43 ± 3.21 **
0.5 Cu	52.87 ± 1.63 **	84.56 ± 0.09 **
1 Cu	80.03 ± 2.17 **	90.07 ± 1.94 **

Compared to 0 Cu scaffold, * *p* < 0.05, ** *p* < 0.01.

## Data Availability

The original contributions presented in this study are included in the article; further inquiries can be directed to the corresponding authors.
